# Neural Mechanisms of Rapid Sensitivity to Syntactic Anomaly

**DOI:** 10.3389/fpsyg.2013.00045

**Published:** 2013-03-18

**Authors:** Albert E. Kim, Phillip M. Gilley

**Affiliations:** ^1^Department of Psychology and Neuroscience, Institute of Cognitive Science, University of Colorado at BoulderBoulder, CO, USA; ^2^Department of Speech, Language, and Hearing Science, University of Colorado at BoulderBoulder, CO, USA

**Keywords:** sentence comprehension, syntactic, anticipatory, prediction, P1, N170, posterior cingulate, occipital temporal cortex

## Abstract

Recent psycholinguistic models hypothesize that anticipatory processing can speed the response to linguistic input during language comprehension by pre-activating representations necessary for word recognition. We investigated the neurocognitive mechanisms of anticipatory processing by recording event-related potentials (ERPs) to syntactically anomalous (*The thief was caught by* *for police*) and well-formed (e.g., *The thief was caught by* *the police*) sentences. One group of participants saw anomalies elicited by the same word in every instance (e.g., *for*; low-variability stimuli), providing high affordances for predictions about the word-form appearing in the critical position. A second group saw anomalies elicited by seven different prepositions (*at*, *of*, *on*, *for*, *from*, *over*, *with*; high-variability stimuli) across the study, creating a more difficult prediction task. Syntactic category anomalies enhanced the occipital-temporal N170 component of the ERP, indicating rapid sensitivity – within 200 ms of word-onset – to syntactic anomaly. For low-variability but not the high-variability stimuli, syntactic anomaly also enhanced the earlier occipital-temporal P1 component, around 130 ms after word-onset, indicating that affordances for prediction engendered earlier sensitivity to syntactic anomaly. Independent components analysis revealed three sources within the ERP signal whose functional dynamics were consistent with predictive processing and early responses to syntactic anomaly. Distributed neural source modeling (sLORETA) of these early active sources produced a candidate network for early responses to words during reading in the right posterior occipital, left occipital-temporal, and medial parietal cortex.

## Introduction

Human sentence comprehension requires recognition of words and integration of those words into larger, combinatory meanings at the rate of two to five words per second. Understanding the neurocognitive mechanisms of this rapid process is a central question for cognitive neuroscience. Models of sentence comprehension have increasingly adopted an anticipatory processing framework, which posits that syntactic and semantic representations of the prior context generate predictions about the future input, allowing faster, more accurate processing of that future input (e.g., Altmann and Kamide, 1999; DeLong et al., 2005; Altmann and Mirkovic, 2009). This anticipatory framework extends and departs from a dominant “feedforward” processing view within psycholinguistic thinking, in which the brain responds to an input with a hierarchically organized sequence of increasingly complex transformations of the input, beginning with low-level sensory and perceptual feature extraction and progressing to higher levels of syntactic and semantic analysis (Frazier and Fodor, 1978; Friederici, 2002). The current work investigates the neural and cognitive mechanisms of sentence comprehension with a focus on the role of anticipatory processing on brain responses within the initial ∼200 ms of the brain’s response to a sentence-embedded words.

In order to understand a sentence, human language comprehenders identify the syntactic categories of words and the syntactic relationships among those categories, allowing very different compositional meanings to be computed for superficially similar strings like *The Republican admired the Democrat* vs. *the Republican was admired by the Democrat* on the basis of syntactic cues. A long tradition of experimental work over the last several decades has indicated that each word in a sentence receives at least a provisional syntactic analysis within a few hundred ms of the word’s arrival at the senses (Ferreira and Clifton, 1986; Trueswell et al., 1994). While it has been uncontroversial that syntactic processing occurs rapidly, the question of how cognitive and neural processes implement such rapid syntactic analysis has been a focus of vigorous debate (MacDonald et al., 1994).

A powerful tool for investigating the neurocognitive mechanisms of real-time syntactic processing is the recording of event-related potentials (ERPs) and their magnetic analog (MEG) during sentence comprehension. ERP and MEG studies report that syntactic anomalies during language processing modulate brain activity within the initial ∼500 ms after stimulus onset, indicating sensitivity to syntactic anomaly. A large number of studies find a late positivity around 500–900 ms in response to syntactic anomaly (Osterhout and Holcomb, 1992; Hagoort et al., 1993; Kim and Osterhout, 2005) and also sometimes a left anterior negativity (LAN) around 300–500 ms (Osterhout and Mobley, 1995; Gunter et al., 1997). A smaller body of work reports strikingly earlier effects, within 200 ms of word-onset (Neville et al., 1991; Friederici et al., 1993). A series of studies of German auditory sentence processing, has found that syntactic category violations (e.g., *Die Kuh wurde im* ***gefüttert***; *The cow was in the* ***fed***^1^), elicit a negative-going deflection in the ERP beginning approximately 150 ms after anomaly onset, which has been termed the early left anterior negativity (ELAN; Hahne and Friederici, 1999; Friederici, 2002).

One interpretation of the electrophysiological data has been that the ELAN effect reflects the activity of a fast syntactic processing mechanism, which recognizes the syntactic category of the incoming word and attempts to assign an initial syntactic representation to this incoming word. The ELAN is attributed to failure to integrate the syntactic category assigned to the incoming word with the developing syntactic analysis of the sentence (Friederici, 2002). Within this model, initial syntactic processing commitments at each word in a sentence are restricted to computations involving the word’s basic syntactic category (e.g., noun, verb, determiner) and not more detailed information about that word (e.g., morphosyntactic details like those that distinguish *eats* from *eat*). This restriction is believed to allow computational efficiencies that speed the initial syntactic analysis. Consistent with this hypothesis, ELAN effects have been observed in a restricted range of situations, which violate constraints on syntactic category (Neville et al., 1991; Hahne and Friederici, 1999), while more fine-grained morphosyntactic anomalies have elicited later effects, on the later LAN and P600 components (e.g., *The boys won’t **eats**/**eat** the food*; Osterhout and Nicol, 1999).

The neural generators of these earliest brain responses to syntactic anomaly are a topic of ongoing investigation. Dipole source estimation of MEG responses to German ELAN stimuli estimated generators in bilateral ventral-lateral prefrontal and superior temporal cortex (Friederici et al., 2000a). An fMRI study using the same stimulus class found BOLD signal mainly in the temporal lobe (Meyer et al., 2000). The fMRI study did not indicate prefrontal sources, diverging from the MEG source estimation data; however, the slow response of the BOLD signal might limit its sensitivity to transient processing events or may not distinguish between early and late responses to a given stimulus (Friederici et al., 2000b). Friederici (2002) has concluded that the left ventral-lateral prefrontal cortex contributes to syntactic structure building, while the anterior aspects of the superior temporal lobe contribute to identifying the syntactic category of incoming words, with both of these contributions occurring rapidly after word-onset, in the time window of ELAN effects.

The speed of the ELAN response has raised the question of how the brain might extract basic perceptual features, identify syntactic category, and distinguish syntactically anomalous from well-formed words, all within ∼150 ms of the onset of the input word (Lau et al., 2006; Dikker et al., 2009). One account of how syntactic category violations can elicit such rapid responses is that syntactic processing generates syntactic *predictions* about an incoming word’s syntactic category, reducing the task of detecting anomaly to identifying the category of the word, rather than one that requires both identifying category and building syntactic structure. Consistent with the idea that predictive processing facilitates early sensitivity to syntactic anomaly, Lau et al. (2006) reports that during English sentence reading, syntactic anomalies elicited left anterior negativities 200–400 ms after the onset of prepositions in positions where a noun was strongly expected (e.g., *Although the bridesmaid kissed Mary*, *she did not kiss Dana’s* ***of** the bride*). This negativity was smaller in situations where expectation for a noun was weaker (e.g., *Although Erica kissed Mary’s mother*, *she did not kiss Dana’s* ***of** the bride*). In this second situation, the possessor *Dana’s* is temporarily compatible with ellipsis (e.g., *Although Erica kissed Mary’s mother*, *she did not kiss Dana’s*), raising the possibility of a well-formed non-noun continuation. The effect pattern was attributed to earlier sensitivity to anomalies that violated a strong prediction of a noun after *Dana’s*, than for anomalies that violated more “open ended” predictions (Lau et al., 2006). It is somewhat unclear how this result relates to the ELAN effect, given that its latency is later than typical ELAN effects.

A variant of the predictive perspective is that syntactic predictions can be imposed on and tested in sensory cortex (Dikker et al., 2009). An MEG study of sentence comprehension in written English found that syntactic anomalies enhanced the M100 MEG component around 130 ms after word-onset. A dipole source for this effect was estimated in low-level visual cortex, in the medial occipital lobe, and not in prefrontal or temporal cortex (Dikker et al., 2009). Given the effect’s localization and its early latency, the authors argue that such responses do not reflect evaluation of syntactic category information but rather the processing of word-form features, which have been pre-activated by top-down predictions, allowing rapid sensitivity to unexpected word-forms. This association of early brain responses to a word – within the initial ∼200 ms after stimulus onset – with sensory-cortical processing is compatible with a number of studies (e.g., Shulman et al., 1997; Tarkiainen et al., 1999; Debener et al., 2003; McCandliss et al., 2003; Kim and Lai, 2012; Kim and Straková, 2012; Mesgarani and Chang, 2012).

A sensory prediction view also potentially accounts for the temporal lobe source estimates for MEG effects to auditory German word-category violations sentences observed by Friederici et al. (2000b). Such sources could involve primary or secondary auditory cortex. Although this study also produced prefrontal sources, the temporal lobe sources had greater dipole strength during the ELAN latency window (Friederici et al., 2000b). Early effects of syntactic anomaly in a mismatch negativity preparation have also produced source estimates consistent with auditory cortex (Shtyrov et al., 2003). Overall, it is possible that the earliest responses to syntactic anomaly reflect predictive processing effects on modality-specific sensory cortex, occurring within occipital cortex during reading and within the superior temporal lobe during listening (Dikker et al., 2009). A sensory prediction account provides an alternative to the possibility that a modality-independent syntactic analysis system rapidly computes syntactic analyses within the initial 200 ms of word recognition. Most studies of the earliest effects of syntactic anomaly have not, however, explicitly manipulated affordances for prediction (though see Lau et al., 2006); thus, it is possible that these rapid responses do not require predictive processing.

The current work used ERPs to investigate the rapid computations of sentence comprehension and the role of anticipatory processing on those computations. Participants saw sentences containing syntactic category anomalies (1b), along with well-formed control (1a) and filler sentences.

1a. *The thief was caught by* ***the** police* … CONTROL.
1b. *The thief was caught by* ***for** police* … ANOMALY.

We examined whether syntactic anomalies would elicit early ERP effects consistent with generators in visual cortex, indicated by ERP scalp distribution and neural source modeling. We furthermore explicitly investigated the role of predictive processing in these early responses by manipulating the affordances for prediction within the stimulus set. For one group of subjects, anomalies were always introduced by the same word (“for”; low-variability stimuli), while a second group of subjects saw anomalies introduced by one of seven different, randomly varying words (*at*, *of*, *on*, *for*, *from*, *over*, *with*; high-variability stimuli). We hypothesized that participants would learn distributional regularities of the experimental sentences within the session, facilitating predictive commitments. In the low-variability stimuli, the smaller range of word-forms encountered at the critical word position provided greater affordances for predicting the word-form in that context, which we hypothesized would lead to faster recognition of critical words and therefore faster sensitivity to the anomaly. The high-variability stimuli created a more difficult prediction task, due to the greater range of items occurring in the critical context, and this may cause greater recruitment of processing resources to the computation of predictions, even as prediction success would be lower.

Unlike the study of Lau et al. (2006), we did not manipulate predictive affordances based on participants’ “long-term” knowledge of the language (e.g., about which contexts allow ellipsis). Instead, we manipulated predictive affordances that could be learned in the “short-term” from the distributional properties of the stimuli within the experimental session^2^. An underlying assumption here is that syntactic predictions are constantly adapted to the current context, a topic we return to in the Discussion.

Our stimuli, by violating restrictions on syntactic category, satisfied the functional antecedents typically hypothesized for ELAN effects (Friederici, 2002).

Unlike most ELAN studies, our experimental design manipulated the nature of the target word (e.g., *the* vs. *for*), while allowing no differences between experimental conditions in the material preceding the target words. Prior ELAN studies manipulated the pre-target material, while holding the target word constant [e.g., … *im* ***gefüttert*** vs. …*im Stall* ***gefüttert*** in Hahne and Friederici (1999)]. Pre-target differences in such studies may affect EEG activity prior to the target word, including the baseline calculation for the critical word, leading to ERP effects in the ELAN time window that are unrelated to the brain response to the target word (Steinhauer and Drury, 2012). We avoided such potential artifactual effects.

We modeled the neural generators of the electrophysiological activity we recorded, with an approach that deviated in potentially critical ways from prior efforts. The prior work used discrete dipole source estimation methods, which require assumptions about the number of discrete dipole sources for the scalp ERP and also initial dipole seed positions. Both of these steps influence the resulting source model. For instance, source modeling of the MEG ELAN-like effect in German (Friederici et al., 2000a) began with an assumption of four dipoles seeded in the left and right lateral-frontal and temporal lobes, based on prior fMRI data (Friederici et al., 2000b; Meyer et al., 2000) and patient data (Friederici et al., 1999), along with a constraint that final solutions would occur within a radius of 10 mm of the seed positions. Source estimation of the M100 effect in English written sentences (Dikker et al., 2009) did not impose this type of constraint on dipole location but did assume a single dipole solution (guided by minimum norm distributed source estimates computed in BESA).

Our approach to source estimation made fewer assumptions about the number, location, or extent of sources of brain activity involved in sentence processing. We used independent components analysis (ICA; Bell and Sejnowski, 1995) to separate the scalp-recorded ERP signal into additive, statistically independent components (ICs), which accounted for substantial portions of the variance between experimental ERP conditions (Gilley and Sharma, 2010). We then subjected each of these ICs to source localization using distributed source models (sLORETA; Pascual-Marqui, 2002), which converge on a pattern of activity distributed across a field of many dipoles within cortex that accounts for scalp-recorded ERP. This “blind” source separation and distributed source estimation approach was motivated by the fact that physiological findings, both from scalp-recorded ERPs and intracranial animal studies, find that visual stimuli are followed by rapid (within ∼200 ms) activation of multiple areas within visual cortex and beyond, suggesting that even at early latencies, neural processing involves a distributed, recurrently connected network of brain regions (e.g., Foxe and Simpson, 2002; Cornelissen et al., 2009). We avoided assumptions – either through the use of a single dipole model or strong constraints on dipole location – that might preclude sensitivity to a distributed network of neural generators for the ERPs observed.

## Materials and Methods

### Participants

Participants were 53 undergraduate students. One participant was excluded from analysis, due to excessive EEG artifact. Twenty-six participants (13 female) were assigned to the low-variability stimuli and were aged 18–28 (mean = 22.3). Another 26 participants (13 female) were assigned to the high-variability stimuli and were aged 18–34 (mean = 22.1). All were right-handed native English speakers, with normal or corrected-to-normal vision, and received course credit for participating. All participants gave informed consent in accordance with the University of Colorado Institutional Review Board.

### Stimuli

We created 70 stimulus sentences, each with a well-formed (1a) and anomalous form (1b) and each seven words long. Well-formed, plausible control sentences consisted of the following sequence: simple noun phrase (e.g., *The thief*), passive verb sequence (e.g., *was caught*), by-phrase (e.g., *by the police*). Anomalous sentences were derived from control sentences by replacing the determiner in the by-phrase (*the*) with a preposition (e.g., *for*). In one version of the stimuli, the anomalous preposition was always *for* (low-variability anomaly). In a second version of the stimuli, seven different prepositions were used to introduce the anomaly (*at*, *of*, *on*, *for*, *from*, *over*, *with*; high-variability anomaly), with stimulus sentences assigned to one of these prepositions.

For each stimulus set (high- and low-variability), two experimental lists were created such that half (35) of the critical stimuli within each list were assigned to the well-formed and anomalous versions, respectively, in a counter-balanced manner. For the high-variability anomaly stimuli, each of the seven anomalous prepositions occurred five times in the list. Across these stimulus sets, word length was controlled: in the high-variability stimuli, the anomalous words were two, three, or four characters long, with an average length of three, which matched the length of low-variability anomalies (“for”) and control stimuli (“the”) at the critical word position.

Stimuli were pseudorandomly interspersed among 105 filler sentences. Thirty-five of the fillers contained syntactic anomalies, containing violations of noun number agreement (e.g., “The woman bought a **cars** …”), pronoun agreement (e.g., “Ralph’s uncle taught **herself** …”), and verb-form (e.g., “The chef will **chopping** …”). The remaining 70 fillers were syntactically and semantically normal. Overall, 70/175 (40%) of the stimuli were anomalous.

### Procedure

Participants were randomly assigned to one experimental list and tested in a single session lasting about 90 min (including about 45 min of experimental preparation). Participants sat in a dimly lit, sound-attenuated booth. Stimuli were presented on an LCD monitor at a distance of 105 cm from the participant, such that words subtended approximately 2°. The participant was instructed to read normally and to try to understand the sentences. Each trial consisted of the following events: a fixation cross appeared in the center of the screen for 600 ms, after which a stimulus sentence was presented one word at a time in the center of the screen. Each word appeared on screen for 300 ms followed by a blank screen interval of 200 ms. Sentence-ending words appeared with a full stop. One-third of the sentences were pseudorandomly followed by a comprehension question about the sentence. The question appeared in the center of the screen and stayed on the screen until one of two buttons on a button box was pressed, indicating an answer of “Yes” or “No.” Participants used their thumbs to respond, with half the participants using their right thumb to answer “yes.” The remaining two-thirds of the stimulus items were followed by a prompt (“PRESS EITHER BUTTON TO CONTINUE”) appearing on the center of the screen until the YES button was pressed. A 1450 ms blank screen interval followed each trial.

### Data acquisition

Continuous EEG was recorded from 64 sintered Ag/Ag-Cl electrodes embedded in an elastic cap (Neuroscan QuikCaps) arranged according to the extended 10–20 system. EEG was amplified and digitized at 1000 Hz (Neuroscan Systems). Vertical eye movements and blinks were monitored with two electrodes placed above and below the left eye, and horizontal eye movements were monitored by electrodes placed at the outer canthi of each eye. EEG was also recorded over left and right mastoid sites. Impedances were maintained below 10 kΩ. EEG was referenced on-line to a vertex electrode and re-referenced off-line to an average reference.

### ERPs

After recording, data was down-sampled to 200 Hz and filtered with a bandpass of 0.1–100 Hz. Eye-blink artifact was corrected using a subject-specific regression-based algorithm (Semlitsch et al., 1986). Any remaining voltages exceeding ±100 μV were rejected, resulting in the loss of 5 and 4% of the trials in the control and violation conditions, respectively. Data from individual channels that contained consistent artifacts within a given participant’s data were replaced by an average of the neighboring channels. ERPs were averaged in epochs of activity spanning −100 to 700 ms relative to the onset of the target stimulus, with voltages quantified relative to a 100 ms pre-stimulus window. Early ERP components (P1 and N170) were quantified for statistical analysis as peak voltages within windows of 125–145 (P1) and 170–270 ms (N170) post-stimulus-onset. These two windows were centered on the peaks of these two components in the grand-averaged data across all experimental conditions. Voltages in these time windows were averaged within four channel-groups: left-posterior (P07, PO5, P7, CB1, O1), right posterior (P08, PO6, P8, CB2, O2), left anterior (F7, F5, F3, FT7, FC5), right-anterior (F8, F6, F4, FT8, FC6). The anterior channel-groups covered electrodes at which left anterior negativities have been observed to syntactic anomalies during sentence processing (Hahne and Friederici, 1999; Lau et al., 2006). The posterior channel-groups included electrodes at which early components elicited by visual words (P1, N170) are typically maximal (Rossion et al., 2003; Maurer et al., 2005). The later N400 and P600 components, which are less “peaky” than the P1 and N170, were quantified as mean voltage within windows of 350–450 ms (N400), and 500–800 ms (P600), which were based on visual inspection of the data and were consistent with prior findings involving these components (e.g. Kim and Osterhout, 2005). Voltages within these windows were averaged within a single central-parietal channel-group (CPZ, PZ, POZ, P1, P2), where N400 and P600 effects are typically maximal (e.g., Kim and Sikos, 2011). These dependent measures (P1, N170, N400, P600) were subjected to repeated measures analyses of variance (ANOVAs). For the P1 and N170, these ANOVAs included a between-subjects factor for variability (high vs. low), and within-subjects factors for sentence-type (anomalous vs. control) and channel-group (left-posterior, right-posterior, left-anterior, right-anterior). The Greenhouse–Geisser (1959) correction for inhomogeneity of variance was applied to all repeated measures with greater than one degree of freedom in the numerator.

### Source estimation

#### Pre-processing

Prior to analysis of the group-level data, we pre-processed the data as follows. For each subject, the EEG was epoched into sentence length trials of 3700 ms, including presentation of all seven words in each sentence. Each epoch was then baseline corrected by subtracting the average of all points in the epoch. ICA was performed across all channels (excluding artifactual channels as described above) of the concatenated trials using the extended Infomax ICA algorithm (Bell and Sejnowski, 1995) as implemented in the EEGLAB toolbox for Matlab (Delorme and Makeig, 2004).

Each IC was then subjected to four separate rejection criteria, resulting in rejection if any one of the criteria were met. First, a correlation was performed between each IC and the voltage fluctuations for each of the eye channels. Components with a correlation value greater than 0.8 and with a back-projected scalp distribution dominating the frontal channels (near the eyes) were treated as eye movement artifacts and removed from further analysis. Second, any component where the maximum weight at a single channel was greater than 3 standard deviations from any other channel was treated as single channel noise and removed from further analysis. Third, percentage of variance accounted for (PVAF) by each component was computed for the full set and compared to the mean variance accounted for (MVAF) computed for the average of all trials. If the MVAF was less than half of the PVAF, then the component was treated as transient artifact and removed from further analysis. Fourth, any component accounting for less than 0.01% of the variance in the full data set was treated as noise and removed from further analysis. Application of these rejection criteria resulted in a mean of 27 retained ICs per subject, which were considered experimentally relevant. Missing channel data was reconstructed separately for each retained IC using a spherical spline interpolation algorithm. Finally, the retained ICs were mixed and projected back to the original channel space.

#### Group-level ICA

In order to identify experimentally relevant sources, an extended Infomax ICA was performed at the group-level on the pre-processed data from each subject, as described above (cf., Congedo et al., 2010). Prior to analysis, each subject’s data was normalized by dividing each point by the maximum absolute amplitude value in the set. Next, all trials from all subjects were concatenated into a group-level data set and subjected to a spatial principal components analysis (PCA). Results of the PCA revealed 21 principal components with an epsilon (variance accounted for) greater than 0.001. ICA was then performed on the rank reduced (rank = 21), group-level data.

Each component was analyzed separately for experimental effects by first projecting each IC to the trial-level of the data. The projected component was averaged across the trials separately for each subject in each level of sentence-type (control versus anomaly). A non-parametric approach was used to test experimental effects, such that we tested for effects of stimulus variability (high-variability versus low-variability), condition effects (control versus anomaly), and for interaction effects via planned comparisons. For each test, 10001 permutations of the data were created by randomly shuffling and dividing the data into comparable sets. A paired *t*-test was used to estimate the experimental distribution for each of the permutations. All probability results were corrected for multiple comparisons using the family wise error rate, and then subjected to a 5-point smoothing across time to reduce spurious effects.

#### Source modeling

Brain source estimations of each IC were computed in Curry 6.1 (Compumedics-Neuroscan), and modeled using the boundary element method (BEM; Fuchs et al., 2002) with a three-shell realistic head volume (scalp, skull, cortex) computed from the standard MNI152 template (Fonov et al., 2009). Source waveforms were computed as the average projection of each IC to the sentence-level data (i.e., the average of all sentence presentations). The signal-to-noise ratio for each IC was computed as the ratio of the largest 20% of the IC amplitudes to the average amplitude of the pre-stimulus baseline (i.e., EEG prior to the first word of each sentence). Sources were estimated using standardized low resolution brain electromagnetic tomography (sLORETA; Pascual-Marqui, 2002) with sources constrained to the cortical volume. Prior research has established that sLORETA on ICA separated data can accurately estimate brain sources, as seen by comparison with intracranial recordings (Van Der Loo et al., 2007). Unlike other distributed source estimations (e.g., minimum norm, LORETA, etc.), the sLORETA algorithm returns statistical test values, in this case *F*-values, which reflect the probability of a source location.

#### Retained components

Results of the group-level ICA and source estimates were combined to select components of interest. Only those components with significant effects and significant source estimations were retained.

## Results

### ERPs

**Figure 1:**
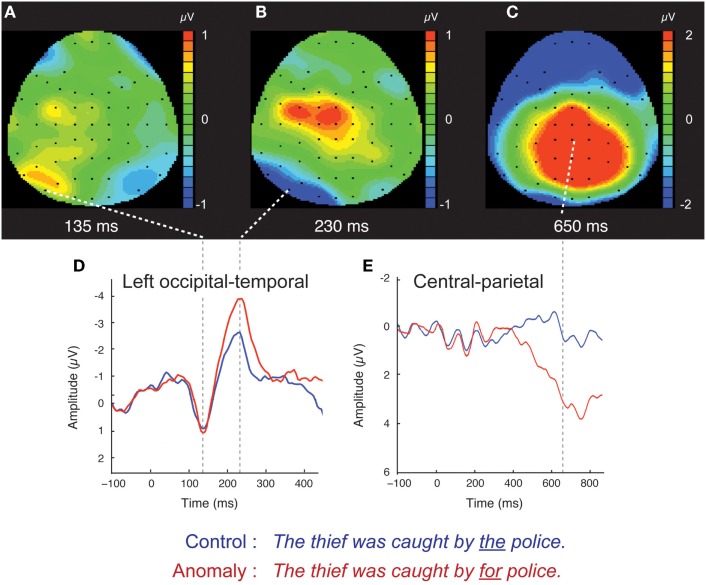
**Scalp ERPs for the syntactic anomaly and control conditions, collapsed across the distinction between high- and low-variability stimuli**. Scalp distributions of the difference between anomaly and control are shown at three time points: 135 ms **(A)**, 230 ms **(B)**, and 650 ms **(C)**. Red (blue) color indicates that anomaly ERPs are positive (negative) going relative to control ERPs. Waveforms for the control and syntactic anomaly conditions are shown for left occipital-temporal channels **(D)**, where the early effects were components were concentrated, and for central-parietal channels **(E)**, where the later effect was concentrated. Scalp ERP waveforms are plotted negative up.

**Figure 2:**
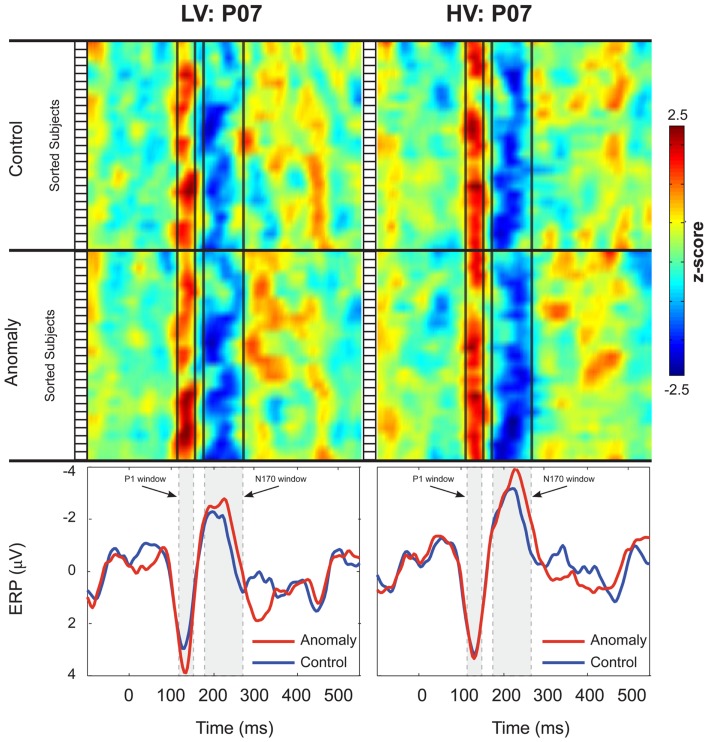
**Inter-subject variability of the P1 and N170 ERPs at electrode P07**. Participants in the Low-Variability (LV) group are shown in the left column, and participants in the High-Variability (HV) group are shown in the right column. *Z*-scores for each participant’s ERPs are plotted as stacked color maps, separated by sentence-type (anomaly and control). The open boxes to the left of each stack represent the boundaries for each subject in the map. Vertical black lines represent the boundaries for the P1 and N170 windows. The bottom panels of each column show the group averaged ERPs for each of the sentence-types. Shaded boxes represent the boundaries for the P1 and N170 windows. Scalp ERP waveforms are plotted negative up.

Both control and anomalous words elicited a positive-going occipital-temporal P1 peak at 135 ms (Figure 1D) followed by a negative-going occipital-temporal N170 peak around 200 ms after stimulus onset (Figure 1D). Inspection of individual participant’s ERPs at a representative left occipital-temporal channel (PO7), indicated that P1 and N170 peak latencies were highly consistent across participants and across levels of stimulus-variability and sentence-type, and that our analysis windows of 125–145 ms (P1) and 170–270 ms (N170) were appropriate for quantifying these components (Figure 2).

**Figure 3:**
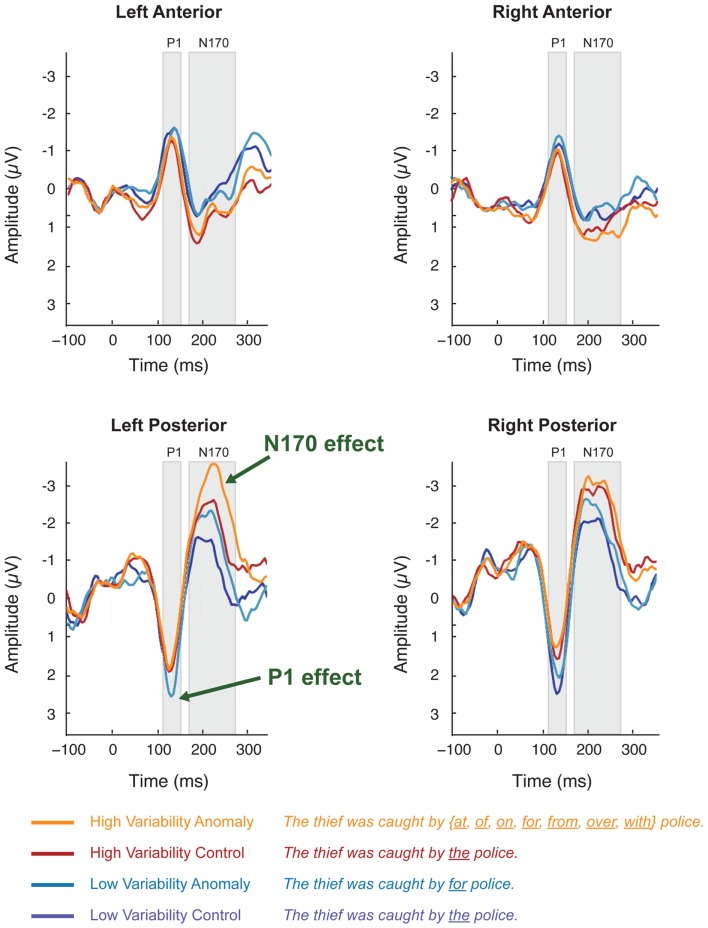
**Scalp ERPs elicited by syntactic anomaly (teal) and control (dark blue) in the low-variability stimuli and in the high-variability stimuli (orange vs. dark red)**. Each plot shows the average of voltages within a five-channel-group: left-posterior (P07, PO5, P7, CB1, O1), right posterior (P08, PO6, P8, CB2, O2), left anterior (F7, F5, F3, FT7, FC5), right-anterior (F8, F6, F4, FT8, FC6). Scalp ERP waveforms are plotted negative up.

The occipital-temporal N170 appeared enhanced by anomalous stimuli, relative to controls (Figures 1B,D and 3). The P1 over left occipital-temporal channels also appeared enhanced by anomalous stimuli, relative to controls, but specifically for low-variability subjects (Figure 3) and not in the grand average data (Figure 1A). At central-parietal sites, anomalous words elicited a large positive shift, relative to controls, beginning around 450 ms and continuing beyond the end of the epoch (Figures 1C,E). Statistical analyses are reported below for the P1, N170, N400, and P600 time windows in the scalp ERP^3^.

#### P1

In the 125–145 ms time window, syntactic anomalies in the low-variability condition elicited more positive peak amplitudes than controls in the left-posterior channel-group. This was reflected in a stimulus-variability × sentence-type × channel-group interaction [*F*(1, 50) = 20.1; *p* < 0.001]. Separate analyses at each channel-group showed that stimulus-variability interacted with sentence-type at the left-posterior channel-group [*F*(1, 50) = 5.11, *p* < 0.05] but not the other channel-groups (*F*’s < 1). Restricting analysis to the left-posterior channel-group, anomalies elicited more positive-going voltages than controls for low-variability stimuli [*F*(1,25) = 6.76, *p* < 0.05], but not for high-variability stimuli (*F* < 1)^4^.

#### N170

In the 170–270 ms time window, anomalous stimuli elicited more negative-going ERPs than the control stimuli, mostly in the left-posterior channel-group, reflected in a sentence-type × channel-group interaction [*F*(1, 50) = 5.83, *p* < 0.05]. This effect did not interact with stimulus-variability, and we therefore collapsed across levels of stimulus-variability in further analyses. Analyses at each channel-group separately showed that anomalous stimuli elicited a negative deflection relative to controls at the left-posterior channel-group [*F*(1, 50) = 5.66, *p* < 0.05]. Other channel-groups showed no effects [*F*’s < 1]. At the right-anterior channel-group, stimulus-variability interacted with sentence-type [*F*(1, 50) = 6.47, *p* < 0.05], reflecting a marginal effect of sentence-type for the high-variability stimuli [*F*(1, 25) = 4.09, *p* = 0.054] but not for the low-variability stimuli.

#### N400

In the 350–450 ms time window, no effects of sentence-type were observed for voltages in the central-parietal channel-group (*F*’s < 1).

#### P600

In the 500–800 ms time window, voltages in the central-parietal channel-group were more positive-going for syntactically anomalous words than control words [*F*(1, 50) = 16.4, *p* < 0.0001]. This effect did not interact with the level of variability.

### Independent components

**Figure 4:**
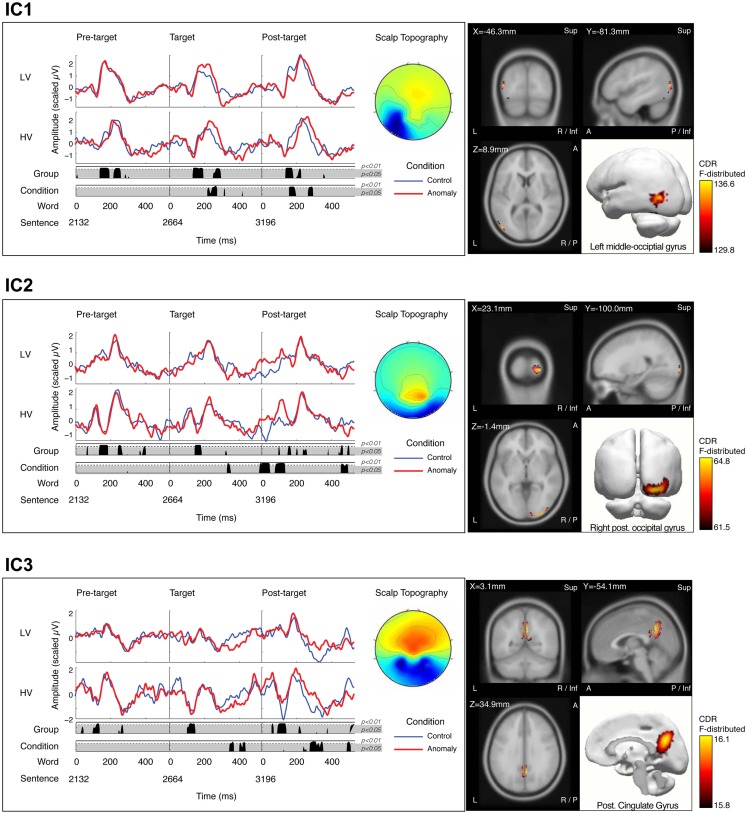
**Three independent components – IC1 (top panel), IC2 (middle panel), and IC3 (lower panel) – demonstrating significant experimental effects**. Each panel contains two primary sub-panels; the left sub-panel showing the mean time series projections of each IC for the final three words of the sentence (pre-target, target, and post-target words, respectively), where the target word is either a well-formed control (blue), or an anomalous form (red). IC projections are shown separately for low-variability stimuli (LV, upper waveforms) and high-variability stimuli (HV, lower waveforms). IC waveforms are shown with positive up, although scalp polarity is determined by the spatial weights for each component. Results of the permutation *t*-tests are plotted below the IC waveforms at each time point, in two rows representing pooled effects of stimulus variability (LV vs. HV; upper row) and pooled sentence-type effects (control vs. anomaly; lower row). Time points with significant differences (*p* < 0.05 shown in the gray shaded region; *p* < 0.01 shown above the dotted line in each row) are shown by the black area plots in the corresponding row. The grand average scalp topography for the IC is plotted to the right of the time series waveforms. The right sub-panel shows the mean sLORETA solution for the IC in four views plotted against the reference MRI (MNI152): coronal, sagital, and axial planes of the MRI (X, Y, and Z, respectively) and rendered as activation in a 3-dimensional model.

Statistical tests of the back-projected, group-level ICs revealed six ICs with group and/or sentence-type effects (*p* < 0.01) that also had significant source estimations. Three of the six components had significant effects during the time range of early processing (∼100–300 ms), while the remaining three ICs had significant effects in the time range of the later N400 and P600 effects. There were no significant effects from the planned comparisons (i.e., interaction effects). Here we report on the network of the three components with early effects; arbitrarily named IC1, IC2, and IC3 (Figure 4).

#### IC1

**Figure 5:**
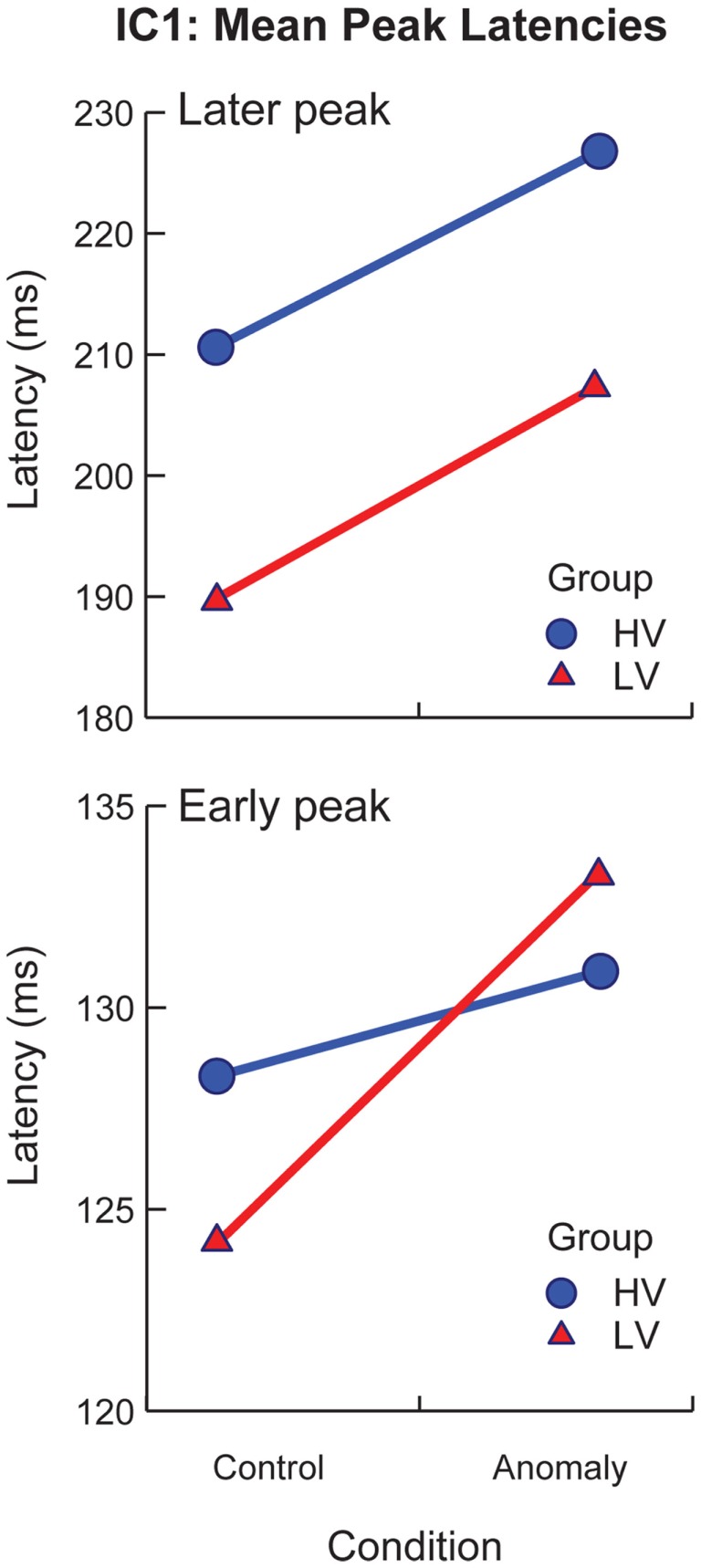
**Mean latencies for peak activation of IC1 by stimulus variability group (low-variability vs. high-variability) and sentence-type (control vs. anomaly)**. Error bars represent standard error.

sLORETA solutions revealed peak activity for IC1 in the left occipital cortex (middle occipital gyrus). The projected time series for IC1 revealed significant group differences (*p* < 0.05, corrected) from 136–196 and 252–296 ms after target onset, and significant effects of sentence-type (*p* < 0.05, corrected) from 220–276 ms after target onset. IC1 contained an early and a later peak around the latencies of the P1 and N170 peaks in the scalp ERP (Figure 4, IC1). Visual inspection of IC1’s mean component projections suggested that the peak latencies of both peaks were modulated by both sentence-type and stimulus-variability. To examine latency effects, the peak latency for IC1 was selected for each subject’s component projection, with separate analyses of the early and the late windows. Latency values were treated as the response variable in a *post hoc* partially repeated measures ANOVA with stimulus-variability (low vs. high) as the between group factor, and sentence-type (control vs. anomaly) as the within group factor. Results of this analysis revealed that the early IC1 peak was delayed for anomaly relative to controls [*F*(1,50) = 28.96, *p* < 0.0001], and this delay was larger for the low-variability than the high-variability stimuli, reflected in a significant Sentence-Type × Stimulus-Variability interaction effect [*F*(1,103) = 9.03, *p* < 0.01]. There was no effect of stimulus-variability on the first peak of IC1 [*F*(1,50) = 0.05, *p* = 0.824]. The second IC1 peak was delayed for anomaly relative to controls, reflected in a significant effect of sentence-type [*F*(1,50) = 54.93, *p* < 0.00001], and also later for high-variability than low-variability stimuli [*F*(1,50) = 7.61, *p* = 0.01], but there was no significant interaction effect [*F*(1,103) = 0.12, *p* = 0.73; Figure 5]. These effects on latency are compatible with increased (continued) activity as prediction demands increase (in high-variability stimuli relative to the low-variability stimuli) and in response to anomaly.

#### IC2

sLORETA solutions revealed peak activity for IC2 in the right posterior occipital lobe (cuneus). The projected time series for IC2 revealed significant group differences (*p* < 0.05, corrected) from 148–188 ms after target onset, and significant sentence-type effects (*p* < 0.05, corrected) at 332–352 ms after target onset. IC2 appears with slightly different temporal morphologies for low-variability and high-variability stimuli. Generally, the waveform morphology reveals a double-peaked activation, peaking at ∼150 and ∼235 ms, respectively. With high-variability stimuli, these two peak activations are separated by a strong negative-going deflection; whereas this deflection is not apparent with low-variability stimuli, as reflected in the group-level differences in the that time range (Figure 4, IC2). Furthermore, this negative deflection is present after each word presentation with high-variability stimuli, suggesting that this deflection is not specific to the target word, but reflects increased prediction-related processing demands throughout the session.

#### IC3

sLORETA solutions revealed peak activity for IC3 in bilateral medial parietal lobe (posterior cingulate gyrus). The projected time series for IC3 revealed significant group differences (*p* < 0.05, corrected) from 104–152 ms after target onset, and significant sentence-type effects (*p* < 0.05, corrected) at 344–372 ms and 404–436 ms after target onset. Similar to IC2, overall activity appears to be larger for high-variability stimuli than for low-variability stimuli. This difference in amplitude is revealed at ∼135 ms post-stimulus after the presentation of each word in the sentence (Figure 4, IC3). Similarly, this finding likely reflects the increased processing demands with high-variability stimuli.

The peak *F*-values for the sLORETA solution of IC3 were significant, but with low *F*-values relative to IC1 and IC2. Given that the scalp topography of IC3 reveals two negative peaks in the distribution (over the left and right hemispheres), it is possible that the sLORETA solutions simply reflect the average space between two mirrored dipoles from more lateral structures. To test this possibility, we implemented two *post hoc* procedures. First, a dipole source analysis was performed under the assumption of two mirrored rotating dipoles with initial seeds at the centroids from the sLORETA solution, but with allowances in final location constrained only to the entire cortical volume. Results of that analysis revealed a best-fit solution at the initial seed points; with more lateral solutions resulting in 95% confidence ellipsoids extending well outside of the head. Second, sLORETA solutions were computed separately for each stimulus-variability-group-BY-sentence-type projection of the component, revealing similar source locations for each tested projection. Taken together, results from the *post hoc* analyses further support the medial parietal lobe as the source of IC3 activity.

In order to explore the question of how well these three IC’s captured the effects observed in the scalp ERP, we conducted a *post hoc* analysis of the early P1 amplitude effect described for the ERP. First, the three selected ICs were mixed and projected back to the trials for each subject, excluding all other ICs. The result of this projection is a “pruned” scalp ERP, containing only IC1-3. Next, the mean ERPs for the left-posterior channel-group were computed, and the peak amplitudes were identified for each subject and sentence-type. The pruned-P1 amplitude values were then subjected to an ANOVA as described above for the channel-groups (see Materials and Methods). Results of that test confirmed the emergence of an interaction effect [*F*(1,50) = 5.09, *p* < 0.05]. While the pruned ERPs did not reveal a significant group effect as reported above, the general trend of an amplitude difference between groups was maintained [*F*(1,50) = 3.35, *p* = 0.07]. Taken together, these findings suggest that the observed interaction effect in the scalp ERP is driven by contributions from (at least) the three identified components.

## Discussion

Syntactic category anomalies during sentence reading elicited rapid brain responses generated within posterior cortex during the initial ∼200 ms after word-onset, modulating the occipital-temporal P1 and N170 components of the ERP. We found no evidence that syntactic category anomalies elicited an ELAN within the initial 270 ms of the response to anomaly. These early responses were followed by the enhancement of a large central-parietal P600, which is the more widely reported effect of syntactic anomaly (e.g., Osterhout and Holcomb, 1992). The early ERP effects of syntactic anomaly were speeded when affordances for predictive processing increased. When anomaly was introduced by the same word throughout the study (low-variability stimuli), syntactic anomalies enhanced the P1 component, relative to controls, around 130 ms post-stimulus onset. Meanwhile, all syntactically anomalous stimuli (low- and high-variability stimuli) enhanced the later N170 component. Source models implicated a network of structures within low- and high-level visual cortex as well as medial parietal lobe, whose activity was sensitive to syntactic anomaly and to prediction demands within the initial 300 ms of processing. The functional response characteristics of these individual sources suggest that they drive the observed ERP effects.

### The earliest ERP responses to syntactic anomaly

Previous studies report that syntactic category violations in auditory sentence comprehension elicit an ELAN. A prominent model of sentence comprehension attributes the ELAN to fast syntactic analysis mechanisms (Friederici, 2002). Although this model focuses on auditory language processing, the same model has sometimes been associated with anterior negativities elicited by word-category violations during reading, suggesting a domain-general rapid syntactic analysis mechanism (e.g., Friederici et al., 1999). The majority of studies of syntactic category violation during reading, however, do not report ELAN effects, raising a question of how general the ELAN effect is across modalities and experimental preparations (cf. Steinhauer and Drury, 2012).

Our results indicate that the earliest ERP responses to syntactic category violations during written language processing are not ELAN effects but rather are posterior in scalp distribution and generated in visual cortex and other posterior cortical structures. Two prior MEG studies have reported similarly early effects generated in visual cortex (Dikker et al., 2009, 2010). Our findings converge with the MEG results (with some discrepancies, discussed below), and deviate from prior ERP literature, which has not previously reported such early posterior effects elicited by syntactic category anomalies. We share the conclusion of Dikker et al. (2009) that the earliest brain responses to syntactic anomaly contain major contributions from modality-specific sensory cortex, resulting in scalp distributions concentrated over occipital-temporal sites during visual sentence processing. Meanwhile, the scalp distribution of ELAN effects during auditory sentence processing is consistent with generators in auditory areas of the superior temporal lobe, although we provide no direct evidence of this last point in the current findings.

Our data demonstrate that predictive processing plays a key role in the earliest brain responses – within the initial ∼170 ms – to syntactically unexpected inputs. When the word-form at the critical word position was easier to predict (the low-variability stimuli), sensitivity to syntactic anomaly began at an earlier latency. The MEG study of Dikker et al. (2009) also concluded that predictive processing drives the earliest responses to syntactic anomaly, but did not directly examine the impact of affordances for prediction. Instead, the early latency (∼130 ms) and source localization (occipital lobe) of the effects were assumed to be incompatible with high-level syntactic analysis and were therefore attributed to top-down predictive commitments. The effect pattern we observe receives no account within the standard ELAN-based model of sentence processing. In this model, the earliest responses to syntactic category anomaly reflect a mismatch between the syntactic category of the incoming word and a rapidly computed phrase-structural grammatical representation (e.g., Friederici, 2002); the model does not explain why the application of syntactic knowledge or assignment of syntactic category should be affected by affordances for prediction.

Our data involve a combination of long-term syntactic knowledge and rapid adaptations of that knowledge in response to recent linguistic experience. The sensitivity of early brain responses (N170) to syntactic anomaly – for both high- and low-variability stimuli – presumably reflects knowledge of the grammatical regularities of the language, accumulated over a lifetime of linguistic experience. The speeding of syntactic anomaly sensitivity under conditions of reduced stimulus-variability (P1 in addition to N170 effects) is compatible with rapid learning of distributional patterns that are specific to the current experiment. We suggest here that such learning engages a general ability to adjust syntactic processing to the diverse linguistic contexts that language users encounter (e.g., specific conversational topics, and speaker-idiosyncracies, and dialects). The learning mechanisms involved here may be the same as those that mediate syntactic priming effects during language production and comprehension (Pickering and Branigan, 1998; Trueswell and Kim, 1998) and which have been associated with incremental learning of syntactic knowledge throughout a linguistic lifetime (Chang et al., 2006).

The rapid learning of local distributional contingencies may have an analog in auditory ELAN findings. In that work, anomalies were consistently introduced by the same word-initial past participle marker in German (“ge,” as in “gefuttert”). The consistency with which this morpheme marked anomaly within the stimuli raises the possibility that participants learn to predict this specific form as an anomaly introducing stimulus, speeding sensitivity to the anomaly.

Although our stimuli were designed to violate constraints on syntactic category, the mechanisms underlying our results are likely not dedicated to syntactic analysis specifically. A number of studies of semantic processing in context report effects on posterior, visual processing ERP components, with early latencies resembling the effects here, suggesting rapid semantic influences on early stages of visual word recognition (Sereno et al., 2003; Dambacher et al., 2009; Kim and Lai, 2012).

Our findings raise a question of why effects similar to our own have not been more widely reported, given the number of previous studies of syntactic anomaly during reading. We suggest that, relative to previous work, the current and related research combine some simple but potent methodological and experimental advances, which improve sensitivity to effects of high-level variables (e.g., syntactic anomaly) on short-latency ERP effects. First, the current study explicitly focuses on early latency ERPs at occipital-temporal sites, where early visual processing effects are most pronounced (Maurer et al., 2005). Many studies have not examined such effects, in part due to an absence of dense sampling over occipital-temporal channels in older recording apparatus and in part due to a priori hypotheses that higher-level linguistic variables will mainly affect the later N400 or P600 components, as has been observed in a large number of findings involving these two components (e.g., Kutas and Hillyard, 1980; Osterhout and Holcomb, 1992). Although a number of recent studies of visual word recognition have focused on early visual processing ERP components (e.g., Bentin et al., 1999), these studies have often presented single words without context, sometimes using shallow tasks (e.g., passive reading). Embedding words in sentence contexts, as we did here, may engage high-level processing and anticipatory commitments more so than isolated word recognition tasks, enhancing sensitivity to high-level processing, such as syntactic analysis. Finally, uncontrolled variability in low-level features such as word length can strongly modulate the earliest brain responses in a way that is much less visible at later components, and obscuring effects of higher-level variables, such as semantic status (Hauk and Pulvermüller, 2004; see also Penolazzi et al., 2007). Therefore, careful control over word length, as implemented here, may be critical for achieving sensitivity to high-level processing effects.

It is not clear how to reconcile the early posterior effects observed here with the minority of studies reporting that syntactic anomalies during reading elicit early anterior negativities resembling the auditory processing ELAN (e.g., Neville et al., 1991). We note here that interpretation of some of these ELAN-like effects during reading is complicated by the possibility that they contain differences between conditions in baseline activity prior to the critical word (Steinhauer and Drury, 2012). The most widely cited example of an ELAN effect during reading is elicited by comparisons like *The scientist criticized Max’s proof* ***of** the theorem. vs. The scientist criticized Max’s* ***of** proof the theorem* (Neville et al., 1991). Here, the critical stimulus word *of* occurs at different sentence positions (one word later in the control than the anomaly condition) and is also preceded by different word-types (a noun vs. a possessor preceded by a noun). Differences between experimental conditions prior to the critical word may affect the baseline calculation, resulting in differences in the ELAN time window that do not reflect the brain’s response to the critical word. In fact, given that all studies reporting ELAN effects to syntactic anomaly involve differences in pre-critical context material (cf. Steinhauer and Drury, 2012), such results must be interpreted with caution.

### An early response network

Independent components analysis extracted three components that accounted for significant portions of variance within the scalp-recorded ERP and also showed significant effects of either sentence-type (anomaly vs. well-formed) or predictability (high- vs. low-variability) within the initial 200 ms of processing at the target word position. Distributed source localization for these three components estimated generators in the left occipital-temporal cortex (middle occipital gyrus), right posterior occipital lobe (cuneus), bilateral medial parietal lobe (posterior cingulate gyrus), respectively. Three additional sources – in the right superior temporal sulcus, right middle temporal gyrus, and left planum temporale – also responded to stimulus-variability and/or sentence-type but at longer latencies, in the time window of the N400 and P600 ERP components; these sources are discussed in a separate report (Gilley and Kim, in preparation).

The three early active sources responded differently to syntactic anomaly and to stimulus-variability (predictability). The occipital-temporal source, but not the other two sources, showed increased activity and a delayed peak to syntactic anomaly, relative to control words, during the initial 200 ms of processing. This sensitivity to anomaly, furthermore, occurred earlier under conditions of low stimulus-variability. These functional properties are consistent with a role in the response to syntactically unexpected words, which is modulated by predictive processing.

All three early active sources exhibited increased activity for high-variability relative to low-variability stimulus words, during the initial 200 ms of processing. The occipital-temporal source additionally peaked later for high-variability than low-variability stimuli. These effects of stimulus-variability occurred not only at the critical word but also at the preceding and following words, during the same early time window. This pattern of effects is consistent with prediction-related processing, which is upregulated by the difficulty of predicting input in the high-variability stimuli relative to the low-variability stimuli. This prediction-related processing appears to be sustained across multiple words within the sentence.

The contributions of the three cortical regions implicated in our findings can be discussed in the context of their experimental responsiveness and existing understanding of these brain structures. The left occipital-temporal generator showed increased activity as prediction became more difficult (high-variability stimuli) and increased activity for syntactically anomalous stimuli relative to controls, suggesting that it is involved in prediction and the evaluation of those predictions. This generator is part of higher-order visual cortex, and may be well-suited to representing visual word-form features. Anticipatory commitments may pre-activate representations within the left occipital-temporal cortex, resulting in lateral-inhibitory conflict when the bottom-up input does not match the pre-activated pattern. The occipital-temporal representations involved here may be related to those implicated in a number of fMRI and patient studies of visual word processing (Dehaene et al., 2005; Price and Devlin, 2011). ERP studies report effects of word recognition and face processing on the occipital-temporal N170 component, which have sometimes been associated with activity in occipital-temporal cortex, although only some of these findings include source localization results (e.g., Rossion et al., 2003). It should be noted that fMRI BOLD effects have overlapped most across studies in the fusiform gyrus, ventral to the source localizations described here. It is possible that the discrepancy between our source localization and these fusiform activations reflects error in sLORETA source estimation and, perhaps to a lesser extent, inaccuracies in fMRI BOLD localization. Alternatively, our sentence stimuli may impose different functional demands from the word recognition tasks that are most common in fMRI studies, resulting in recruitment of related but distinct regions of the ventral visual stream. Understanding potential differences in the contributions of lateral vs. more ventral regions of occipital-temporal cortex to language processing is an issue for future research. However, consistent with any further illumination of this issue is a core conclusion that predictive commitments about visual word recognition during sentence comprehension may be imposed on and evaluated in higher-order ventral visual cortex, in the left hemisphere.

Posterior occipital cortex showed increased activity for high-variability stimuli, consistent with upregulation by prediction demands, but did not show early sensitivity to syntactic anomaly (Figure 4, IC2). We suggest that perceptual representations in posterior occipital cortex undergo preparatory pre-activation, like those in occipital-temporal cortex – reflected in the effect of stimulus-variability. However, the representations in posterior occipital cortex encode lower-level visual features, which do not systematically discriminate expected from unexpected words in the situations examined here. Previous MEG studies indicate that posterior occipital cortex is sensitive to basic visual distinctions such as foveal position and spatial extent at ∼100 ms (e.g., Tarkiainen et al., 1999). Such low-level features were highly controlled in the current experiment (e.g., control and anomaly stimuli were matched for average length).

Whereas our results indicate that the earliest sensitivity to syntactic category violations occurs in lateral occipital cortex, Dikker et al.’s (2009, 2010) MEG studies reported sensitivity at a similar latency localized to posterior occipital cortex, medial to our posterior occipital source. These different source localizations might reflect distinct response properties of posterior and downstream ventral visual system areas, such as lateral occipital-temporal cortex. In the Dikker et al. (2009) results, anomaly was correlated with word length, and sensitivity to this low-level feature may underlie the effects (Dikker et al., 2009). In the findings of Dikker et al. (2010), category-violating words elicited M100 responses whose amplitudes correlated with the degree to which their phonological properties were atypical for the licensed syntactic category – as quantified by a statistical analysis of the relationship between phonological features and syntactic categories (Farmer et al., 2006). This effect was interpreted as evidence that predictions about grammatical category result in predictions about low-level visual features in posterior occipital cortex, leading to sensitivity to category violations. The types of visual cortical representations that underlie such sensitivity remain poorly understood, and it is not clear what distinguishes such sensitivity from the sensitivity to word-forms that we associated here with lateral occipital cortex. Future work could systematically manipulate the correlation between syntactic anomaly and course-grained word-shape features like length or phonological word-class typicality and investigate the relative involvement of posterior occipital vs. occipital-temporal cortex^5^.

Medial parietal cortex also showed increased activity for high-variability stimuli, indicating that this area is part of the network of prediction-related language processing structures. Medial parietal cortex has been activated in previous fMRI studies of syntactic processing (Kuperberg et al., 2003) and narrative processing (Yarkoni et al., 2008).

However, the contribution of medial parietal cortex to language understanding remains little explored. Critically, this area is not a primary or even secondary sensory area (Parvizi et al., 2006). fMRI studies have recently implicated medial parietal lobe, including posterior cingulate and surrounding retrosplenial cortex, in the processing of contextual associations (Bar and Aminoff, 2003; Bar et al., 2007) which mediate predictive cognitive commitments (Bar, 2007). The medial parietal cortex is interconnected with both medial temporal lobe (MTL) and thalamus (Parvizi et al., 2006), and has been associated functionally with “assigning mnemonic associations to sensory input” (Vogt et al., 1992). We suggest that the medial parietal lobe’s known connectivity and functional properties render it well-situated to modulate sensory processing based on predictions generated from MTL mnemonic representations of syntactic and semantic knowledge. Within a linguistic processing context, medial parietal cortex may serve as a hub for implementing perceptual predictions about linguistic forms based on recent context.

### On the viability of rapid, recurrent processing

The conclusions here posit rapid recurrent interactions between visual cortex and higher-order representations encoding syntactic knowledge – within ∼200 ms post-stimulus onset. Such conclusions contrast with a number of previous ERP and MEG studies suggesting that prior to ∼200 ms, the visual system is still in the early stages of a feedforward sequence of low-level feature extraction. For instance prior studies reported that brain responses before ∼200 ms distinguish alphabetic character strings from non-alphabetic stimuli but do not distinguish among alphabetic stimuli on the basis of semantic properties or lexical status (Nobre et al., 1994; Bentin et al., 1999; Tarkiainen et al., 1999; Pylkkanen et al., 2002; Mariol et al., 2008; Solomyak and Marantz, 2009). These findings might indicate that the visual system has extracted visual features that respond to word-like stimuli but has not yet accessed higher levels of representation. In the context of such findings, it is worth addressing whether rapid recurrence is physiologically viable and why we observe it here, where other studies do not.

We note here that a fast timeline for feedforward and recurrent information flow within the system is compatible with a substantial body of physiological data, even if the prior language processing ERP literature has not pointed clearly to this conclusion. Human ERP studies find that occipital cortex responds to visual stimuli by 56 ms and that frontal cortex is active by 80 ms (Foxe and Simpson, 2002). Monkey intracranial recordings show that feedforward information flow from V1 to the highest levels of the ventral visual system (inferotemporal cortex, IT) occurs in ∼23 ms (Schroeder et al., 1998, 2001) and that robust selectivity for complex stimuli (e.g., faces) occurs at latencies of ∼100 ms (Rolls and Tovee, 1994). A number of studies indicate that transmission time for information flowing along a single synaptic distance is 10–15 ms, both between and within cortical regions (Tovee, 1994). Thus, the latency of our anomaly related effects includes sufficient time for feedforward information flow from V1 forward to multi-modal integration areas of the brain and even recurrent interaction among lower- and higher-order brain regions. Once information has arrived at higher-order areas, numerous feedback projections, which outnumber feedforward projections in the brain (Felleman and Van Essen, 1991), provide routes for recurrent information flow.

## Conclusion

Normal language processing is embedded in contexts, which offer rich affordances for anticipation. An anticipatory processing perspective has fundamental implications for how we understand the flow of information within among sensory and non-sensory brain mechanisms during language processing. Syntactic knowledge, stored in memory and modulated by recent context, can project predictive influences onto all layers of analysis. In the case of “early,” perceptual representations of an incoming word, such anticipatory processing can pre-activate relevant representations before they are needed. This leads to rapid recognition when the input matches expectations and also rapid sensitivity when the input deviates from expectations. Within this approach, relatively low-level responses can participate in high-level computations, through interaction with higher-order representations.

## Conflict of Interest Statement

The authors declare that the research was conducted in the absence of any commercial or financial relationships that could be construed as a potential conflict of interest.

## Footnotes

^1^A past participle (e.g., *gefüttert*) is atypical following the word *im* (“in the”), although not illegal (Steinhauer and Drury, 2012). A noun is much more likely, as in baseline sentences like *Die Kuh wurde im Stall **gefüttert** (The cow was in the barn **fed***).

^2^It is possible that the findings of Lau et al. (2006) also reflect short-term learning of contingencies within their stimuli, such as the fact that a number of stimuli within the experiment involve ellipsis. However, here, we explicity assume that this occurs.

^3^A numerical difference between anomaly and control waveforms was also visible within the initial 100 ms of processing (see, for example, Figure 2, bottom left). However, statistical analysis of mean voltages in a 60–80 ms latency window found no significant differences between anomaly and control voltages.

^4^For both the P1 and N170 time windows, we also performed ANOVAs on mean voltage within the time windows 125–145 ms (P1) and 170–270 ms (N170), which produced qualitatively identical effect patterns to those report for the peak amplitude analysis here.

^5^We also consider it possible that differences in source estimation methodology and recording technique (EEG vs. MEG) may account for some of the difference in source localizations observed here and by Dikker et al. (2009, 2010). This is also an issue for additional investigation.
